# A143 PATIENT POSITION AND ENDOSCOPIC CHOLANGIOPANCREATOGRAPHY (ERCP) TECHNICAL SUCCESS AMONG PATIENTS WITH SURGICALLY ALTERED FOREGUT ANATOMY: A RETROSPECTIVE ANALYSIS

**DOI:** 10.1093/jcag/gwad061.143

**Published:** 2024-02-14

**Authors:** M Scaffidi, K Khalaf, K Pawlak, D Chopra, D Tham, S B Malipatil, N Gimpaya, B Chan, E Yeung, N Forbes, N Calo, A Mokhtar, C Na, J Mosko, G May, S Grover

**Affiliations:** Medicine, Queen's University Faculty of Health Sciences, Kingston, ON, Canada; St Michael's Hospital, Toronto, ON, Canada; St Michael's Hospital, Toronto, ON, Canada; St Michael's Hospital, Toronto, ON, Canada; Faculty of Health Sciences, McMaster University Faculty of Health Sciences, Hamilton, ON, Canada; St Michael's Hospital, Toronto, ON, Canada; St Michael's Hospital, Toronto, ON, Canada; Scarborough Health Network, Scarborough, ON, Canada; Scarborough Health Network, Scarborough, ON, Canada; University of Calgary Cumming School of Medicine, Calgary, AB, Canada; St Michael's Hospital, Toronto, ON, Canada; St Michael's Hospital, Toronto, ON, Canada; St Michael's Hospital, Toronto, ON, Canada; St Michael's Hospital, Toronto, ON, Canada; St Michael's Hospital, Toronto, ON, Canada; St Michael's Hospital, Toronto, ON, Canada

## Abstract

**Background:**

Patients with surgically altered gastrointestinal anatomy undergoing endoscopic retrograde cholangiopancreatography (ERCP) pose challenges due to anatomical distortions. Factors such as patient positioning, endoscopist experience, and choice of endoscope may influence procedural success. It is unclear how these factors may impact the technical success of ERCP among patients with altered anatomy.

**Aims:**

We primarily aimed to determine the impact of patient positioning (prone versus left lateral decubitus [LLD]) on technical success of ERCP among patients with surgically altered anatomy. Our secondary aim was to determine the impact of patient positioning on procedural time and immediate bleeding.

**Methods:**

We conducted a retrospective single-centre study using data from 2010 to 2020 that included patients with hepaticojejunostomy, Roux-en-Y anastomosis, Billroth-1, or Billroth-2 anatomy. The primary outcome was technical success of the ERCP, which we comprehensively defined as of successful navigation to the papilla or surgical anastomosis, selective cannulation and cholangiography, and the realization of the intended therapeutic goals. The secondary outcomes were the presence of immediate bleeding and procedural time. Statistical analysis involved descriptive statistics using mean and standard deviation (SD) and Fisher exact test with relative risk (RR) and 95% confidence interval (95% CI). All statistical tests were two-tailed and considered significant at Pampersand:003C0.05.

**Results:**

Among 205 patients, there were 179 (87.3%) in the LLD group, and 26 (12.6%) in the prone group. There were no statistically significant differences between the two groups in terms of patient sex, age, type of altered anatomy, or American Society of Anesthesiologist (ASA) classification. We found that there was no significant differences between the two groups in terms of procedural success (RR 1.1, 95% CI: 0.8-1.5), immediate bleeding (RR 1.7, 95% CI: 0.2-14.8), and procedural time (P=0.808).

**Conclusions:**

We did not find that patient positioning had a significant impact on technical success in ERCP among patients with surgically altered anatomy. The choice of positioning should be tailored to individual patient and endoscopist factors, with a focus on optimizing outcomes in this complex patient subset.

**Funding Agencies:**

None

